# Repression of Androgen Receptor Transcription through the E2F1/DNMT1 Axis

**DOI:** 10.1371/journal.pone.0025187

**Published:** 2011-09-26

**Authors:** Conrad David Valdez, Joanne N. Davis, Hana M. Odeh, Tristan L. Layfield, Craig S. Cousineau, Thomas R. Berton, David G. Johnson, Kirk J. Wojno, Mark L. Day

**Affiliations:** 1 Department of Urology, University of Michigan Comprehensive Cancer Center, University of Michigan, Ann Arbor, Michigan, United States of America; 2 Science Park-Research Division, Department of Carcinogenesis, University of Texas MD Anderson Cancer Center, Smithville, Texas, United States of America; The University of Texas M.D Anderson Cancer Center, United States of America

## Abstract

Although androgen receptor (AR) function has been extensively studied, regulation of the AR gene itself has been much less characterized. In this study, we observed a dramatic reduction in the expression of androgen receptor mRNA and protein in hyperproliferative prostate epithelium of keratin 5 promoter driven E2F1 transgenic mice. To confirm an inhibitory function for E2F1 on AR transcription, we showed that E2F1 inhibited the transcription of endogenous AR mRNA, subsequent AR protein, and AR promoter activity in both human and mouse epithelial cells. E2F1 also inhibited androgen-stimulated activation of two AR target gene promoters. To elucidate the molecular mechanism of E2F-mediated inhibition of AR, we evaluated the effects of two functional E2F1 mutants on AR promoter activity and found that the transactivation domain appears to mediate E2F1 repression of the AR promoter. Because DNMT1 is a functional intermediate of E2F1 we examined DNMT1 function in AR repression. Repression of endogenous AR in normal human prostate epithelial cells was relieved by DNMT1 shRNA knock down. DNMT1 was shown to be physically associated within the AR minimal promoter located 22 bps from the transcription start site; however, methylation remained unchanged at the promoter regardless of DNMT1 expression. Taken together, our results suggest that DNMT1 operates either as a functional intermediary or in cooperation with E2F1 inhibiting AR gene expression in a methylation independent manner.

## Introduction

Androgens are required for prostate gland development and for prostatic function and glandular maintenance in the adult male [1]. Androgen action is mediated through the androgen receptor (AR), a ligand-activated nuclear transcription factor. AR expression is found in a variety of tissues, including prostate and breast, and changes throughout development, aging and malignant transformation (reviewed in [2]). AR exists in the cytoplasm and is associated with at least three heat shock proteins, hsp56, hsp70 and hsp90 [3]. Upon androgen binding and activation of the AR, heat shock proteins dissociate and expose a nuclear localization domain which directs the receptor to the nucleus [4]. At the nucleus, the androgen/AR complex undergoes dimerization and phosphorylation prior to nuclear translocation and subsequent binding to androgen response elements (ARE) in the promoter or enhancer region of numerous androgen-responsive genes. Several AR co-activators have been identified (ARA 70, ARA 55 and ARA 54) which also interact with and regulate AR gene transactivation [5], [6]. Thus, ligand-activated AR may regulate genes through a variety of mechanisms. AR function and the signaling pathways regulated through androgen and AR interaction have been extensively studied for decades; however, regulation of the AR gene itself is not clearly understood.

Transcriptional regulation of AR is cell specific and age-dependent [7], [8]. The promoter region of the AR gene lacks transcriptional regulatory sequences (TATA and CAAT), but is rich in GC sequences [9], [10]. There are at least two transcription initiation start sites whose use vary depending on cell type [11]. Studies of the AR promoter have identified potential binding sites for several transcription factors, however, there have only been a few well characterized studies demonstrating transcriptional regulation of AR. For example, Sp1 [9], [11] has been shown to be a positive regulator of AR gene expression, whereas, NF-κB p50/p50 and NF-1 have been shown to be strong negative regulators of AR [12], [13]. The mechanisms underlying the repression of the AR gene remain to be elucidated.

The E2F family of transcription factors control cell proliferation by regulating cell cycle progression [14], [15], [16]. The E2F family has eight characterized family members (E2F1-E2F8) which can form heterodimers with DP family members (DP1, 2, and 3), giving rise to functional E2F activity [16]. E2Fs control entry into the cell cycle and regulate G1/S phase transition by regulating the transcription of genes that encode cell cycle regulatory proteins including Cyclin E, Cyclin A, Cdc 2, Cdc 25A, and proliferating nuclear cell antigen (PCNA), as well as enzymes involved in nucleotide biosynthesis such as dihydrofolate reductase, thymidylate synthase and thymidine kinase [17]. E2F1, E2F2 and E2F3 are traditionally thought of as transcriptional activators of E2F responsive genes, whereas E2F4, E2F5, E2F6, E2F7, and E2F8 act as transcriptional repressors. Overexpression of E2Fs 1–3 in serum starved cells induces S-phase entry and DNA synthesis by binding to DNA response elements and activating the transcription of E2F target genes [17], [18], [19]. E2Fs 1–3 can also override growth-arrest signals induced by Cdk inhibitors p16 [19] and can act as both oncogenes and tumor suppressors [20], [21], [22]. E2F1 binding sites have been reported in the promoters of the breast cancer susceptibility gene BRCA1 [23], p73 [24], [25], the tumor suppressor gene p14^ARF^ [26], and the gene for apoptosis protease-activating factor 1 (Apaf-1) [27]. We have identified E2F binding sites in the promoter of DNA methyltransferase 1 (DNMT1) that allow for regulation by E2F1 [28]. E2F1 has also been shown to act as a direct transcriptional repressor for several genes including urokinase-type PA (uPA) [29], the anti-apoptotic protein Mcl-1 [30], and human telomerase reverse transcriptase [31]. These results suggest that E2F1 can have both positive and negative regulatory roles on gene transcription, however the molecular basis of these disparate functions is not known.

The DNA methyltransferases (DNMT 1, 3a, 3b and 3L) play an integral role in the epigenetic regulation of many genes. All DNMTs except for 3L have a catalytic domain that facilitates the transfer of methyl groups from S-adenosyl-methionine to cytosines located in CG dinucleotides. DNMT 3a and 3b are generally responsible for genome-wide *de novo* methylation during early embryogenesis [32]. DNMT3a is shown to methylate both maternally and paternally imprinted genes in germ-line cells [33], while DNMT3b maintains the chromosomal stability of 6, 9, and 16 via centromeric methylation [34]. Genomic methylation by DNMT3 (a and b) is enhanced in the presence of DNMT3L [35]. Initial methylation by the DNMT3 family members is maintained by DNMT1, which has an affinity for hemi-methylated DNA during replication and cell division. DNMT1 is necessary for mouse fetal development and the progenitor and self-renewing characteristics of somatic cells located in the epidermis [36]. As mentioned, DNMT1 was characterized in our lab to be a direct transcriptional target of E2F1 [28] and may mediate targeted repression by E2F1.

In this study, we explored a regulatory mechanism that controls the endogenous expression of AR in prostate epithelium. We investigated the effects of the transcription factor E2F1 on AR mRNA and protein expression in both human and mouse prostate epithelial cells. We demonstrate how E2F-1, a classical transcriptional activator, might cooperate with DNMT1 to repress AR transcription in the prostate gland.

## Results

### Transgenic K5-E2F1 prostate glands exhibit hyper-proliferative epithelium and an atypical morphology

**Figure 1:**
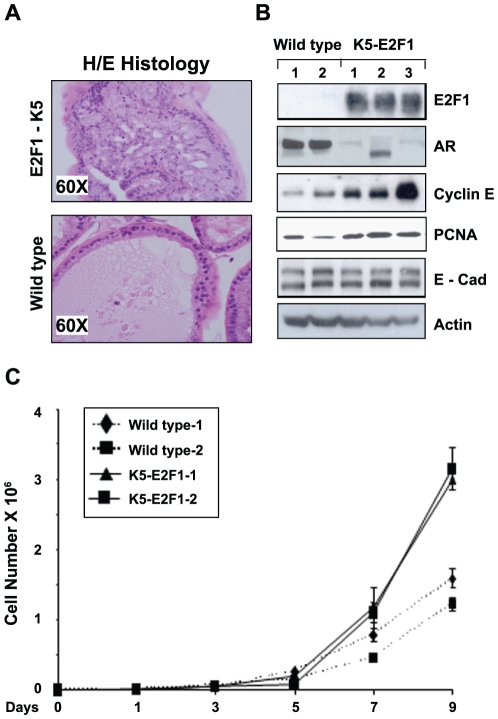
E2F1 leads to atypical prostatic morphology and increases prostate epithelial proliferation in a K5-E2F1 transgenic mouse. *(A)* Histology of prostate tissue taken from both K5-E2F1 transgenic and wild type mice. *(B)* Prostate epithelial cell lines established from both transgenic and wild type mice were analyzed by western blot for the expression of E2F1, cell cycle genes (Cyclin E and PCNA), the epithelial cell specific marker E-cadherin (E-cad), and Androgen Receptor (AR). Actin is shown as a loading control. *(C)* A trypan blue exclusion assay was implemented to measure the viability of cell lines obtained from the mouse models. Each point represents the mean of three independent experiments with the standard deviation.

Accumulating evidence suggests that increased E2F1 activity reactivates several aspects of benign and malignant disease including increases in cellular proliferation [37], [38]. We have shown previously that normal human prostate gland expresses low levels of E2F1 [39]. We observed that keratin 5 promoter driven expression of the human E2F1 gene in the mouse prostate gland [40], resulted in hyperproliferative changes that were not detected in wild type mice (Figure 1A). This K5 promoter fragment is known to direct transgene expression to the basal cell compartment of stratified epithelia of several glandular tissues such a mammary gland, salivary gland and prostate [40], [41]. In K5-E2F1 transgenic mice, the majority of glands appeared grossly normal and were lined with a single layer of epithelial cells, however there were focal areas of increased epithelial hyperplasia with abnormal gland architecture in the dorsolateral lobe of the prostate (Figure 1A). Some glands had increased stratification of epithelial cells that formed compact glands with a cribriform growth pattern, representative of prostatic intraepithelial neoplasia (PIN) and some nuclear atypia. Similar lesions were not detected in wild type animals. Prostate tissue from age and strain-matched wild type mice consisted of normal prostatic ducts lined with a single layer of epithelial cells surrounded by a thin layer of stroma (Figure 1A). To further define a role for E2F1 in prostate epithelial cell growth, we generated prostate epithelial cells lines from glands harvested from two wild type mice and three K5-E2F1 transgenic mice. Semi-quantitative RT-PCR analysis using specific primers for mouse and human E2F1 show the presence of endogenous mouse E2F1 in wild type and K5-E2F1 cells, however, human E2F1 was only detected in K5-E2F1 cell lines (data not shown). In agreement with the hyper-proliferative epithelial histology, the K5-E2F1 lines exhibited a 2 fold increase in cell viability compared to wild type cells (Figure 1C). We also observed a significant reduction of K5-E2F1 cells in G1 phase and a concurrent increase in the distribution of cells in G2/M and S phase (data not shown). Western blot analysis reflected E2F1 expression and revealed a significant increase in human E2F1 protein expression in all three K5-E2F1 transgenic lines compared to wild type controls (Figure 1B). To investigate the molecular events associated with increased E2F1 expression, we analyzed several regulators of prostate epithelium in addition to E2F1 target genes. Whole cell lysates prepared from log phase wild type and K5-E2F1 cells were analyzed for Cyclin E and PCNA protein levels. K5-E2F1 cells exhibited an approximate 3 fold increase in Cyclin E and a 2 fold increase in PCNA (Figure 1B). Cyclin E and PCNA are E2F1 target genes that regulate DNA synthesis and promote G1/S transition, suggesting that E2F can control both DNA replication and mitotic activities in our transgenic prostate model and cell lines [42], [43]. The prostate epithelial lineage of these cell lines was verified by the expression of the epithelial cell marker E-cadherin (Figure 1B) and the steroid hormone receptors estrogen receptor-beta (ER-β) (data not shown). All 3 K5-E2F1 lines exhibited significant repression of AR protein compared to the wild type cells (Figure 1B).

### E2F1 down regulates AR expression and the promoter activity of AR target genes

**Figure 2:**
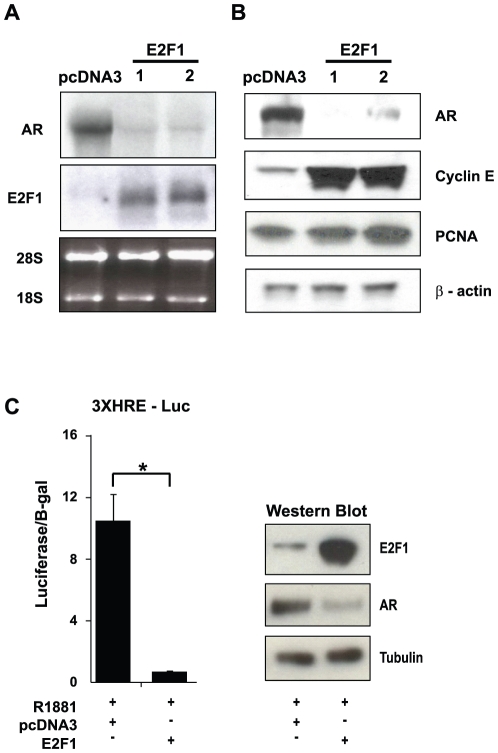
Exogenous E2F1 inhibits both AR expression and responsive promoters. (*A)* Northern blot analysis of stably transfected mouse prostate epithelial cells (PrE) with either pCDNA3 (control vector) or E2F1 shown as E2F1-1 and E2F1-2 to detect AR and E2F1 gene transcription. The 28S and 18S ribosomal bands are shown for loading comparison. (*B)* Western blot analysis of whole cell lysates harvested from PrE cells stably transfected with pcDNA3 (control) or E2F1 for the detection of AR, Cyclin E, and PCNA. β-actin is shown as a loading control. (*A and B)* The northern blot and western is representative of 3 separate experiments. LNCaP cells were co-transfected with 1 µg of ARE-Luc (*C)* and 500 ng of either empty pcDNA3 vector or E2F1 in the presence of 10^−9^ M R1881. Results were normalized to β-galactosidase (B-gal) from a co-transfected CMV promoter driven B-gal reporter construct. The histogram represents the mean value of three independent experiments with the indicated standard deviation. The western depicts the expression levels for E2F1 and AR relative to tubulin for the transfection and treatment conditions. * indicates P<0.05 for the indicated comparison in brackets.

To determine if E2F1 directly represses AR transcription, we examined whether exogenous expression of E2F1 reduces AR mRNA levels in prostate epithelial cells. Stable E2F1 over-expressing clones were established in mouse prostate epithelial cells (PrE) and two clones, PrE2F1-1 and PrE2F1-2, were expanded and characterized. Total RNA was harvested and subjected to Northern blot analysis for the detection of AR and E2F1 mRNA. Both clones exhibited increased E2F1 mRNA and significantly reduced AR mRNA (Figure 2A) and protein (Figure 2B). These cells exhibited increased E2F activity by exhibiting increased expression of Cyclin E and PCNA; two well described E2F-target genes (Figure 2B). These result demonstrated that exogenous E2F1 is involved in the repression of AR expression.

The findings from the transgenic animals indicated that E2F1 might be driving a proliferative and undifferentiated phenotype. We had previously observed the AR-regulated prostate specific antigen (PSA) gene was down regulated following E2F1 over expression suggesting a repressive activity of E2F1 on AR target genes through the repression of AR [39]. To explore this possibility, we examined the effect of E2F1 on a hormone-responsive promoter/reporter construct (3XHRE-Luc) in the androgen-responsive prostate cell line, LNCaP. The 3XHRE-Luc construct has 3 hormone response elements cloned in front of a luciferase reporter gene and allows for the monitoring of directed hormone receptor activation. LNCaP cells were co-transfected with the 3XHRE-Luc construct with either pcDNA3 (empty vector) or E2F1. We treated cells with the synthetic androgen R1881 to specifically activate AR and observed activity from the 3XHRE-Luc reporter (Figure 2C). Co-transfection of E2F1 both abrogated 3XHRE-Luc activity in the presence of R1881 and down regulated AR protein expression (Figure 2C). These results demonstrate that E2F1 inhibits transcriptional regulation of AR target gene promoters by inhibiting AR expression.

### Transcriptional repression of AR requires the transcriptional regulatory domain of E2F1

**Figure 3:**
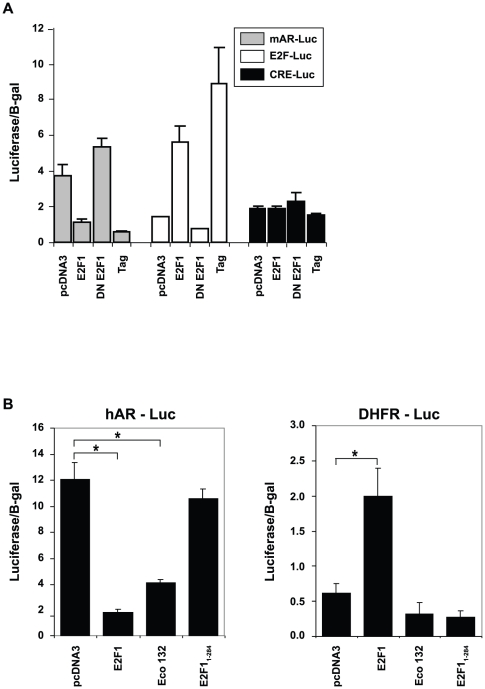
The E2F1 transactivation domain is required for AR promoter activity repression. (*A)* mPrE cells were co-transfected with 1 µg of mARp-Luc, E2F-Luc or CRE-Luc luciferase reporter constructs with 0.5 µg of empty vector (pcDNA3), wild type E2F1, dominant negative E2F1 (DN E2F1) or SV-40 Large T antigen (Tag). After 72 hours, cells were harvested and assayed for luciferase expression. The results are shown as averages of three independent experiments. Assays were done in triplicate and mean values are shown with standard deviation. The results were normalized to β-galactosidase (B-gal) expression from a co-transfected CMV promoter driven B-gal reporter construct. *(B)* LNCaP cells were co-transfected with either 1 µg of hAR-Luc or DHFR-Luc and 0.5 µg of either wild type E2F1or E2F1_1–284_ mutant constructs. The histograms represent the mean value of three independent experiments with the indicated standard deviation. Results were normalized to β-galactosidase (B-gal) expression from a co-transfected CMV promoter driven by a B-gal reporter construct. * indicates P<0.05 for the indicated comparison in brackets.

To determine if E2F1 exerts repressive activity directly on the AR promoter, we utilized a 1.5 kb (−1571 to +131 bp) mouse AR promoter construct (Figure 3A) cloned upstream of a luciferase reporter cassette (mAR-Luc). This plasmid was co-transfected into normal mouse prostate epithelial (mPrE) cells with either empty pcDNA3 vector (control) or wild type E2F1 along with a CMV promoter-driven β-galactosidase (B-gal) reporter plasmid as an internal control. Wild type E2F1 reduced mouse AR promoter activity 3.5 fold compared to cells transfected with empty pCDNA3 plasmid (Figure 3A). To assess AR promoter activity resulting from the direct disruption of E2F1 activity, we used a dominant negative E2F1 (DN E2F1) construct encoding a fusion cassette of the E2F1 DNA binding and the Rb pocket domain. This fusion binds to E2F consensus regions and blocks endogenous E2F1 activity at E2F1-responsive promoters [44] and when employed in our system relieved repression of the AR promoter. As a control, we demonstrated that E2F1 activates an E2F-inducible promoter containing 4 adjacent E2F consensus binding sites (E2F-Luc), while the DN E2F1 construct repressed promoter activity. We also demonstrated that E2F1 does not have an effect on an unrelated CRE-Luc promoter, which contains 4 adjacent cyclic AMP regulatory elements in front of a luciferase reporter construct. We demonstrated that exogenous E2F1 repressed AR promoter activity. To assure that endogenous E2F1 carried out this repressive activity, we disrupted the inhibitory effect of endogenous Rb on E2F1 by co-transfection with SV-40 large T antigen (Tag) and assessed AR promoter activity. Ectopic expression of Tag led to 9 fold activation of the E2F-Luc promoter, but repressed mAR promoter activity nearly 4 fold (Figure 3A). These results confirm that E2F1, normally a transcriptional activator, participates in the repression of the AR promoter.

To elucidate the mechanism of E2F-mediated inhibition of AR, we examined the effects of two functional E2F1 mutants on AR promoter activity. A mutation in the DNA binding domain (Eco 132) failed to significantly relieve E2F-mediated inhibition (Figure 3B). However, deletion of the transactivation domain of E2F1 (E2F1_1–284_) abrogated the inhibitory effect of E2F1 on the AR promoter (Figure 3B). As expected, these E2F1 mutants did not activate the dihydrofolate reductase-luciferase reporter construct (DHFR-Luc), which is known to require both E2F1 transactivation and DNA binding domains (Figure 3B). These results indicate that the transactivation domain of E2F1 appears to be more essential than the DNA binding domain for E2F1 repression of the AR promoter. This observation prompted us to examine co-repressive factors that are involved in the E2F1 mediated repression of AR.

### DNMT1 down regulation relieves AR repression in AR negative cells lines

**Figure 4:**
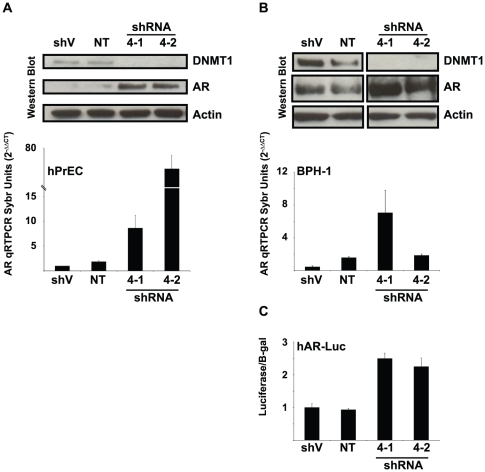
DNMT1 downregulation relieves AR Expression. (A) Primary cultures of human prostate epithelial cells (hPrEC) and (B) immortalized benign prostate epithelial cells (BPH-1) transduced with either control (shVector (shV) and shNon-targeting (NT)) or DNMT1 shRNA constructs. AR and DNMT1 protein expression relative to actin loading control were analyzed by Western blot. The exposures in (B) were taken from different sections of the same blot at the same intensity. AR transcription was analyzed using qRTPCR with readings done in triplicate (graphs A and B). Mean values are represented with standard error bars. (C) shRNA transduced BPH-1 cells (described in A and B) were transfected with the human AR promoter luciferase reporter (hAR-Luc) construct. The histograms represent the mean value of three independent experiments with the indicated standard deviation. Results were normalized to β-galactosidase (B-gal) expression from a co-transfected CMV promoter driven B-gal reporter construct. All western blots are representative of 3 separate experimental replicates.

We have previously shown that the DNA methyltransferase 1 (DNMT1) gene, which typically functions to maintain the methylation and repression of specific genes, was trans-activated by E2F1 [28]. Interestingly, DNMT1 may also exist in a repressive complex that includes E2F1 [45]. To determine if DNMT1 is involved in E2F1 dependent repression of AR transcription, we assessed whether AR expression is relieved in the AR negative defined human primary prostate epithelial cells (hPrEC) following DNMT1 shRNA knockdown. The hPrEC line is a model of transit/amplifying cells of the prostate gland and as such lacks markers of terminal differentiation such as AR expression [46], [47]. These cells allow for the study of normal mechanisms that regulate AR expression. The hPrEC line was subjected to a transient transduction with either empty short hairpin RNA (shRNA), vector, non-targeting shRNA or DNMT1 targeting shRNA (4-1 and 4-2) (Figure 4A) and processed for both qRTPCR and western blot analysis. Compared to controls the expression of the DNMT1 shRNA sequence resulted in a significant decrease in DNMT1 expression at both the transcriptional (data not shown) and protein level (Figure 4A). AR protein and transcription increased in response to decreases in DNMT1 expression, indicating that gene repression may also involve DNMT1. To assess the role of DNMT1 on AR promoter activity, we cloned a region of the AR gene containing a 2 kb human AR promoter upstream of a luciferase reporter (hAR-Luc). Because primary hPrEC cells cannot withstand multiple passages required for stable shRNA transduction, we employed the immortalized human prostate epithelial line, BPH1, which still maintain a non-transformed phenotype [48]. The hAR-Luc construct along with a CMV promoter-driven β-galactosidase internal control reporter were co-transfected into BPH-1 cells that were previously transduced with DNMT1 shRNA targeting constructs (Figure 4B). DNMT1 shRNA relieved AR promoter activity in BPH-1 cells when compared to controls (Figure 4C). These results suggest that DNMT1 contributes to the repression of AR promoter activity in normal prostate epithelial cells.

### DNMT1 associates with the intronic and minimal promoter regions of the AR gene independent of methylation activity

**Figure 5:**
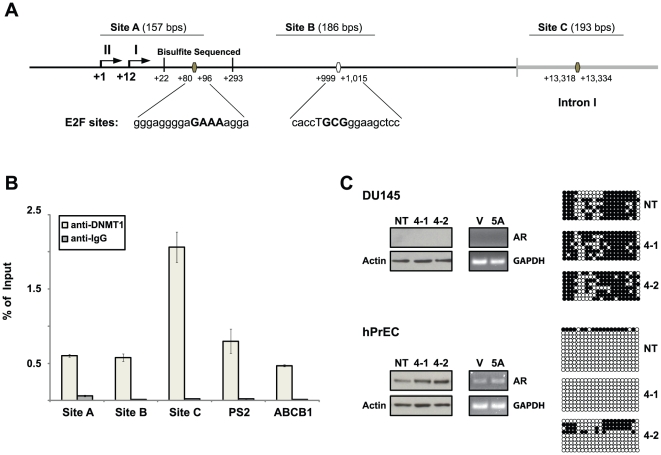
Methylation independent association of DNMT1 with the AR gene. (A) Map of a region of the AR promoter depicting two types of E2F consensus sequences +80→+96 and +13,318→+13,334 (shown as solid ovals) and +999→+1,015 (shown as open ovals). Primer flanked regions are designated as sites A, B, and C. The 243 bp region (+22→+293) analyzed by bisulfite sequencing is indicated. (B) qPCR analysis of target (DNMT1) and non-specific (IgG) ChIPed DNA from BPH-1 cells using primers that flank sites A, B, and C. Primers flanking a region in the PS2 (targeted DNMT1) promoter and ABCB1 (non-targeted DNMT1) region were used as ChIP controls. Data is representative of the mean from 3 qPCR reactions and shown as a percent of input with the standard error indicated. (C) Bisulfite sequencing analysis of the 243 bp region in the AR minimal promoter in hPrEC compared to DU145 cells infected with both non-targeting (NT) (control) and DNMT1 shRNA constructs (4-1 and 4-2). Solid circles (methylated) and open circles (un-methylated) were used to represent the methylation status of cytosines within CpG dinucleotides. Each horizontal strand of circles depicts a separate DNA clone. Lysates were probed on a western blot for AR and actin. Cell lines were also treated with either 1 µM 5Aza (5A) or DMSO matched vehicle (V) and extracted cDNA was PCR amplified with both human AR and GAPDH. All data shown except for the 5Aza treatments are representative of 3 separate experimental replicates.

To understand how DNMT1 functions to represses AR expression, we explored the possibility for DNMT1 to physically associates with the AR gene. Chromatin immunoprecipitation sequencing (ChIP-seq) analysis identified both DNMT1 and E2F1 associated regions across the whole hPrEC genome (data not shown). H-peak analysis [49], [50] of the data revealed regions in the AR genomic structure exhibiting significant DNMT1 and E2F1 co-occupancy. Considering that DNMT1 has been reported to form complexes that bind to E2F responsive promoters, we designed primers flanking specific E2F consensus sequences (site A, B, and C) in the AR promoter. Site A and B were located within 1,000 bps of the transcription start site, while site C corresponded to a location in the ChIP-seq identified region of DNMT1 and E2F1 co-occupancy in the first intron (Figure 5A). Although the ChIP-seq demonstrated some E2F1 associations with the AR gene, we focused our ChIP analysis on DNMT1 interactions, considering that the region under analysis presented with weak E2F consensus sites and that the E2F1 DNA binding domain was not necessary for AR promoter repression (Figure 3B). Primers were used to analyze a known binding target of DNMT1 located in the PS2 promoter [51] and a non-related DNA sequence located in exon 2 of the ABCB1 gene [52]. Rabbit IgG was used to control for any non-specific DNA binding incurred by the antibodies. Quantitative PCR indicates that DNMT1 strongly associates with intronic region showing a ≥4-fold over non-specific enrichment at the ABCB1 genomic region. DNMT1 demonstrated some association with sites A and B in the AR gene, showing slightly increased levels of enrichment over ABCB1, that were similar to amplification levels at the PS2 promoter (Figure 5B). DNMT1, therefore, associates with the 5′ UTR and a region in the first intron of the AR gene that has a possible affinity for E2F1.

DNMT1 is traditionally thought to facilitate the repression of target genes through a catalytic process that involves the transfer of methyl groups to cytosines located in CG dinucleotides present in the DNA sequence. Aberrant hyper-methylation of the AR promoter has been detected in the AR negative metastatic prostate cancer cell lines DU-145 and TSU-PR1 [53]. ChIP analysis demonstrated that DNMT1 associated with a section of DNA spanning a region (+44 to +54) of heavy methylation conserved between DU145s and other transformed AR lacking cell lines [54]. To determine whether methylation of the AR minimal promoter associated region (Figure 5A) is dependent on DNMT1, we sequenced a section (+22–+293) of bisulfite converted DNA extracted from DU145s and hPrECs infected with DNMT1 shRNA. The methylation pattern remained unchanged in the absence of DNMT1 when compared to the cells infected with the non-targeting shRNA construct (NT) in DU145s, while subtle increases were observed in a single hPrEC DNMT1 knockdown cell line (Figure 5C). According to this data, methylation at the AR minimal promoter appears to mostly occur in a DNMT1 independent fashion. We additionally demonstrated that AR expression resulting from the down regulation of DNMT1 occurred regardless of methylation at the bisulfite sequenced region (+22–+293) in hPrECs. To further asses the possibility for a methylation independent process we treated both DU145 and hPrEC lines with a global DNA methylation inhibitor, 5-aza-2′-deoxycytidine (5-Aza) and observed no change in AR transcription (Figure 5C). These data point to a possible mechanism of AR repression in normal prostate epithelial cell lines that utilizes methylation - independent DNMT1 activity.

## Discussion

In this study we have shown that E2F1 either drives the expression of or cooperates with DNMT1 to repress AR transcription in normal undifferentiated prostate epithelium. Specifically, exogenous E2F1 down-regulated AR promoter activity and mRNA and protein expression, while a dominant negative E2F1 construct (DN-E2F1) relieved AR promoter repression by inhibiting access of endogenous E2F1 to E2F targeted promoters. All of these observations correlated with variations in activation of the E2F-target gene promoter DHFR-Luc, and changes in expression levels of endogenous cell cycle regulatory proteins consistent with E2F1 activity. The use of E2F1 functional mutants suggests that the transcriptional regulatory domain of E2F1 comprises this repressive activity possibly through the interaction of an intermediary co-repressor. Based on studies showing that DNMT1 is both a target [28] of E2F1 and that it may co-repress some targets with E2F1 [45], we decided to evaluate the role of DNMT1 in AR repression. Targeted knockdown of DNMT1 relieved AR promoter activity, mRNA and protein expression. Additionally, DNMT1 directed ChIP showed association of DNMT1 with the AR promoter. The lack of de novo methylation at the minimal AR promoter following loss of DNMT1 suggests DNMT1 represses AR expression in a methylation independent manner.

Sharma et al. have recently demonstrated that E2F1 transactivates the AR gene on a depleted RB1 background in an engineered model of castrate resistant prostate cancer [55]. This observation is interesting in light of our findings in non-transformed prostate epithelium in which E2F1 represses AR transcription in the presence of functional pocket proteins. Our findings that the Large T antigen facilitates E2F1-mediated repression of AR, suggests that E2F1 has roles in both the activation and repression of AR transcription. While Sharma et al. provide evidence for a mechanism of AR activation that involves the association of E2F1 with specific regions of the AR promoter; our results did not find association of E2F1 with these reported sites, but revealed regions exhibiting weak E2F1 consensus binding downstream of the AR transcription start site that demonstrated DNMT1 association instead. The only common site studied between our two groups revealed a lack of E2F1 binding coupled with no AR activation, but moderate DNMT1 binding associated with AR inhibition. These seemingly contradictory findings might begin to explain how E2F1 functions in a more traditional role to activate AR in the absence of RB in prostate cancer cells, yet represses AR transcription in normal (non-transformed and non-immortalized) prostate epithelium in the context of functional RB.

**Figure 6:**
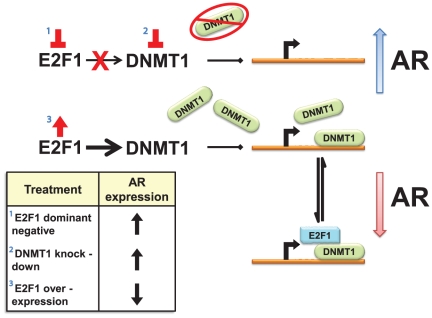
Schematic representation of AR repression through the E2F1/DNMT1 axis. The use of either a dominant negative E2F1 construct or a shRNA to knock down DNMT1 both result in the downregulation of DNMT1 and subsequent rescue of AR expression. E2F1 overexpression experiments suggest that elevated E2F1 levels increase DNMT1 protein expression that associates directly with the AR promoter or possibly in complex with E2F1 to repress AR.

Although E2F1 is thought to primarily function as a positive regulator of transcription, a negative regulatory role of E2F1 has also been described for a number of genes [29]. Unlike its positive regulatory function, which is mediated by the direct interaction of E2F1 with DNA, the mechanism(s) for E2F1-mediated negative regulation are still largely unknown. Crowe et. al. identified two putative E2F binding sites in the hTERT promoter that were important for E2F1- mediated repression [31]. The E2F1 mutant (E132) which lacks the DNA binding domain of E2F1 was inefficient at repressing hTERT promoter activity, suggesting that the DNA binding domain was essential for repression. In another study, direct repression of the Mcl-1 promoter by E2F1 also required the DNA binding domain, but not the transactivation domain [30]. Koziczak et. al. demonstrated that both the DNA and transactivation binding domains of E2F1 were necessary for the negative regulation of uPA and the PA inhibitor (PAI-1) genes [29] however, E2F repressed promoter activity independently of the pocket protein Rb. We have shown here that E2F1-mediated repression independent of Rb pocket protein family members, suggesting that multiple mechanism(s) exist for E2F1-mediated repression. We noted that the AR promoter does not contain a strong E2F1 consensus binding site (TTTGCGG/CG/CAAA), furthermore the E2F1 DNA binding domain was not required for repression of the AR promoter suggesting that E2F1 does not bind directly to the AR promoter, but cooperates with other regulatory proteins to repress AR. Our data demonstrate that the carboxy-terminal transactivation domain was essential for E2F1 suppression of the AR promoter (Figure 3) and therefore supports two possible models of AR repression. Several proteins are known to bind to this region and regulate transcription including CREB-binding protein [56], MDM2 [57] and TRRAP/Tip60 complex [58]. E2F1 may regulate AR promoter activity by forming a known repressive complex that includes Rb, HDAC1, and DNMT1 through an as of yet undefined domain. The transactivation domain also interacts with the basal transcription factor IIH (TFIIH) [59] which may facilitate the E2F1 contacts necessary to induce transcription at the DNMT1 promoter, increasing DNMT1/AR gene interactions that lead to repression (Figure 6). Our findings along with previous work in the lab support a linear model of AR repression that is reliant on the positive transcription of DNMT1 by E2F1, however, the possibility remains for E2F1 to regulate AR expression through a complex involving DNMT1 (Figure 6).

We have shown through targeted knockdown of DNMT1 and ChIP analysis that the association of DNMT1 with weak E2F consensus sites in the AR gene inhibits transcription. A previous study has shown that estrogen receptor (ER) re-expression at both the transcriptional and protein level, results from the targeted inhibition of DNMT1 in ER negative breast cells [60]. DNMT1 is traditionally thought to cause genetic repression through methylation; however, the current understanding of methylation facilitated repression continues to evolve in the field of epigenetics. Glypican 3 (GCP3), a developmental associated gene, is regulated by a promoter methylation independent mechanism in human fetal systems [61]. Methylation sensitive restriction digests show that methylation at the GPC3 promoter is sex specific and occurs regardless of GPC3 expression status in females, but remains absent in males. Yakabe et. al. demonstrated through ChIP analysis that methyl CpG binding protein 2 (MeCP2), which is usually dependent on DNA methylation for genetic interaction, was able to both associate with unmethylated promoter sequences and regulate the expression of a subset of selected genes [62]. Furthermore DNMT1 was reported to repress p21 and BIK in a methylation independent manner [63]. Epigenetic regulation possibly involves the intercommunication of multiple epigenetic marks to orchestrate the regulation of genetic expression. The simultaneous employment of ChIP and methyl specific primer (MSP) analysis, verified the presence of transcription promoting histone modifications (acetyl-H3K9 and dimethyl-H3K4), associated with unmethylated regions responsible for facilitating hTERT expression from a heavily methylated promoter in cancer cells [64]. The role of methylation independent DNMT1 regulation at the AR promoter may involve other epigenetic modifications.

DNMT1 appears to function in concert with other factors to regulate gene expression. Heterochromatin protein 1 (HP1) GST fusions pull down DNMT1, 3a, and 3b [65]. Smallwood et. al. and colleagues further demonstrated that methylation by DNMT1 in complex with the HP1 proteins was dependent on G9a H3K9me2 using *in vitro* chromatin array methylation assays. DNMT1 is also known to directly interact with the enhancer of zeste 2 (EZH2) protein, which mediates H3K27me2/3, in the context of the Polycomb repressive complexes (PRC2/3) [66]. Furthermore, DNMT1 was shown to associate with histone deacetylase activity during direct interaction with HDAC1 [67] and in complex with HDAC2 and corepressor DMAP1 [68]. DNMT1 is likely to facilitate repression at the AR promoter through the interaction with a multi-subunit complex.

Clearly, mechanisms for AR amplification and mutation play a role in prostate cancer progression, however, loss of AR has been reported in a subset of hormone-independent cancers, including a complete loss in some cases [69], [70]. Highly proliferative cells that present with an AR negative phenotype are actually necessary for normal prostate development. Prostate stem cells differentiate into an AR negative transit-amplifying (TA) population that is known to transiently undergo multiple rounds of cellular division before terminally differentiating into AR positive luminal epithelium [47]. Certain prostate cancers may present with mutations that allow for unhindered TA cell proliferation as mentioned in a review by Paul C. Marker [71]. Our findings demonstrate that exogenous E2F1 inhibited activation of the AR responsive promoter construct, 3XHRE-Luc in a minimally invasive cell line, and that the repression is possibly mediated by methylation independent DNMT1 activity at the AR promoter in TA cells.

We suggest that the repression of AR may facilitate a proliferative state in normal transit amplifying basal cells. However, androgens are generally understood to promote epithelial proliferation in AR–responsive cell lines, such as LNCaP and in the regenerating prostate gland following castration. Some suggest that androgens may contribute to re-expanding cellular populations by directly activating androgen receptors expressed in the epithelium. While studies demonstrate that the proliferation of the developing epithelium is supported by the secretion of growth factors from AR activated stromal cells [72], Waltregny et. al. observed proliferation in a population of AR positive luminal epithelial cells during androgen induced prostate regeneration [73]. However, the authors demonstrated that luminal cells additionally expressing p27 failed to proliferate in the epithelium. AR activation by androgens is also thought to drive an anti-proliferative program in the in prostate epithelium as well. Directed expression of functional AR in both malignant and non-transformed AR negative human cell lines (PC-3 and BPH-1) reduced proliferation following androgen stimulation [74], [75]. Tam et. al. demonstrated that the anti-proliferative effect of melatonin on minimally transformed prostate epithelial cells requires the transcriptional upregulation of p27kip1 by AR [76]. The lack of AR expression in prostate epithelial cells may therefore be associated with proliferation as knockout of AR in mouse prostate epithelium resulted in epithelial hyper-proliferation [77]. Mechanisms of AR repression may contribute to epithelial proliferation and although the role of E2F1 in regulating prostate epithelial growth is still not fully understood, we propose that the inhibition of AR expression by the E2F1/DNMT1 axis may be required for normal growth of specific basal cell population in the prostate gland.

## Materials and Methods

### Cell Culture

LNCaP and DU145 cell lines obtained from American Type Culture Collection, Rockville, MD and BPH-1 cells received from Dr. Simon Hayward, Vanderbilt University Medical Center [48] were cultured in RPMI 1640 medium supplemented with 8% fetal bovine serum (FBS), 0.1% penicillin/streptomycin and 0.1% L-glutamine. Human prostate epithelial cells (hPrEC) purchased from Lonza/Clonetics, Walkersville, MD were maintained in Prostate Epithelial Cell Growth Medium (Lonza/Clonetics). Mouse prostate epithelial (PrE) cells, previously described in reference [78] were cultured in RPMI 1640 medium supplemented with 5% fetal bovine serum (FBS), 0.1% penicillin/streptomycin and 0.1% L-glutamine.

For the development of the PrE-E2F1 stable cell lines, PrE cells were transfected with pcDNA3-E2F1 (kindly provided by W. Kaelin [79], or empty vector pcDNA3 (Invitrogen, Carlsbad, CA) using Tfx50 (Promega, Madison, WI) according to the manufacturer's protocol. Stable clones were selected in RPMI 1640 media containing 5% FBS, 0.1% penicillin/streptomycin, 0.1% L-glutamine and 200 µ/ml G418 (Sigma, St. Louis, MO). For the development of the DNMT1 knock-down stable BPH-1 and transient hPrEC cell lines, cells were lentivirally infected with a pLKO.1-puro vector either expressing DNMT1 specific short hairpin RNA (shRNA) (clone ID: NM_001379.1-1687s1c1), non-targeting shRNA (Cat#: SHC002) or no shRNA insert (Sigma/Mission, St. Louis, MO). Stable shRNA BPH-1 clones were selected in RPMI 1640 media containing 8% FBS, 0.1% penicillin/streptomycin, 0.1% L-glutamine and 1 µg/ml puromycin (Sigma, St. Louis, MO).

### Cell treatments

All synthentic androgen (R1881) treatments were done at 10^−9^ M for 24 h after 15 h of serum starvation. All 5-aza-2′-deoxycytidine (5Aza) (Sigma) treatments were done in either complete culturing media used for DU145 or hPrEc lines. Fresh media containing 1 µM 5Aza was added every 24 h for a total of 72 h and DMSO treatments were matched as vehicle controls.

### Northern Blot Analysis

Total RNA was prepared using QIAGEN RNA Easy kit per manufacturer's protocol (QIAGEN, Valencia, CA). Twenty micrograms of RNA was resolved by gel electrophoresis under denaturing conditions and RNA was transferred to a Duralon-UV membrane (STRATAGENE, La Jolla, CA) overnight by capillary action in 20× SSC buffer (3 M NaCl and 0.3 M Na Citrate). RNA was crosslinked to the membrane by UV cross linking. A 1.6 kb human AR cDNA fragment was isolated from CMV3-hAR3.1 (kindly provided by D. Robins, University of Michigan, Ann Arbor, MI) using a HindIII and NheI restriction enzyme sites. A 1.5 mouse AR cDNA fragment was isolated from CMV5-mAR (kindly provided by D. Robins, University of Michigan, Ann Arbor, MI) using HindIII restriction enzyme sites. The human and mouse AR cDNA fragments were gel purified using QIAGEN Gel Purification Kit, per manufacturer's protocol and subsequently labeled with [α-^32^P] dATP using the random oligonucleotide-primer labeling kit (STRATAGENE) and purified on STRATAGNE Nucleotide Push Columns following manufacturer's protocol. The [α-^32^P] dATP labeled probes were hybridized to a Duralon-UV membrane (STRATAGENE) at 65°C overnight in hybridization buffer (0.25 M Na_2_HPO_4_, pH 7.2 and 7% SDS) while rotating. The membrane was subsequently washed twice for 45 min each in 20 mM Na_2_HPO_4_, pH 7.2 and 5% SDS followed by two additional washes for 45 min each in 20 mM Na_2_HPO_4_, pH 7.2 and 1% SDS. The membranes were exposed to X-ray film (Kodak, Rochester, NY) overnight and visualized by autoradiography.

### Western Blot Analysis

Cells were either trypsinized, centrifuged and washed one time with PBS then lysed with RIPA buffer (50 mM Tris pH 8.0, 120 mM NaCl, 0.5% Nonidet P40, 1.0 mM EGTA, 200 µg/ml PMSF, 50 µg/ml aprotinin, 5 µg/ml leupeptin, 200 µM sodium orthovanidate) or lysed directly in the plate. The hPrEC lines were harevested 4 days post infection, while stably infected BPH-1 cells were collected 4 days post selection in puromycin (1 µg/ml). Protein concentrations were determined using Bradford Protein Assay Reagent (BIO-RAD, Hercules, CA), following manufacturer's protocol. For Western blot analysis, protein extract was subjected to gel electrophoresis either on a Tris-glycine polyacrylamide gel (Invitrogen) (LNCaP and mPrE) or on a Nu Page 3–8% Tris-acetate polyacrylamide gel (Invitrogen) (BPH-1 and hPrEC). The gel was transferred to Optitran nitrocellulose membrane (Schleicher & Schuell Biosciences Inc. Keene, NH) by electrophoresis for 1 hour at 45 V. The membrane was blocked in 10% nonfat dry milk in TBST (10 mM Tris, 250 mM NaCl, 1% Tween 20) for 1 hour at room temperature and immunoblotted with primary antibodies for AR (N-20, Santa Cruz, Santa Cruz, CA), E2F1 (KH-95, Pharmingen, Franklin Lakes, NJ), Rb (Pharmingen), PCNA (C-20, Santa Cruz), E-Cadherin (H-108 Santa Cruz), Cyclin E (M-20, Santa Cruz), DNMT1 (Raw M0231S prep gift from Dr. Sriharsa Pradhan, New England BioLabs Inc., Ipswich, MA), β-actin (C-11, Santa Cruz), or actin (AC-40 Sigma, St. Louis, MO). The membrane was incubated with secondary antibody conjugated to horseradish peroxidase (BIO-RAD, Hercules, CA) and the bands were detected using ECL (PIERCE, Rockford, IL) detection system, following manufacturer's protocol.

### Luciferase Assay

LNCaP and PrE cells were plated at 2×10^5^ cells per 6 well dish and incubated at 37°C overnight. Stably infected BPH-1s with DNMT1 shRNAs were plated at a 1 to 60 passage into a 12 well dish and incubated at 37°C overnight. Cells were co-transfected with 1 µg/ml of either of the following promoter-luciferase reporter constructs; DHFR-Luc, E2F-Luc and CRE-Luc were kindly provided by G. Denis, Boston University, Boston, MA [80], 2.0 kb human AR promoter-Luc (hAR-Luc) (kindly provided by F. H. Sarkar, Wayne State University, Detroit, MI), 1.5 kb mouse AR promoter-Luc (mAR-Luc) was kindly provided by D. J. Tindall, Mayo Clinic, Rochester, MN [81], MMTV-Luc (gift from E. Keller, University of Michigan, Ann Arbor, MI) and 3XHRE-Luc (gift from D. Robins, University of Michigan, Ann Arbor, MI). The promoter-reporter constructs were co-transfected in LNCaP and PrE cells with either empty pcDNA3 vector, wild type E2F1 or the following E2F1 mutants (E2F_1–284_, or Eco132) (gifts from W.D. Cress, Moffitt Cancer Center, Tampa, FL, [30], [82]), a dominant negative E2F1 was kindly provided by W. Kaelin, Harvard University, Boston, MA, [44] or Tag (gift from M. Imperiale, University of Michigan, Ann Arbor MI). The pSV-beta-galactosidase (β-gal, Promega) expression plasmid was co-transfected into LNCAP and PrE cell lines at 0.1 µg/ml and into hPrEC and BPH-1 cells at 1 g/ml as an internal control. DNA was transfected using Tfx50 transfection reagent (Promega) at a ratio of ∼3∶1 (Tfx50: DNA) following manufacturer's protocol. After 72 hours of transfection, whole cell lysates were collected in lysis buffer. Luciferase expression was determined by adding 50 µl luciferase substrate (Promega) to 50 µl of lysate and luciferase was monitored using a Monolight 2010 luminometer. B-gal expression was monitored using B-gal Detection System (Tropix, Bedford, MA) following manufacturer's protocol using Monolight 2010 luminometer. Samples were assayed in triplicate and luciferase activity was normalized to B-gal activity.

### qRTPCR and PCR Analysis

Total RNA was extracted by scraping and collecting cells in TRizol (Invitrogen, Carlsbad, CA) (1 ml per 60 mm dish). The lysate was added at 1 ml to a pre-spun 2 ml heavy phase lock gel tube (5 PRIME Inc., Gaithersburg, MD), incubated for 5 min at room temperature, and combined with chloroform. After the mixture was centrifuged at 12,000×g for 10 min at 4°C, the resulting aqueous mixture above the wax plug was removed and mixed together with 500 µl of isopropanol, and incubated for 10 min at room temperature. The mixture was centrifuged into a pellet at 12,000×g for 10 min at 4°C and washed 1 time in 70% ethanol. RNA was reconstituted in 35 µl of UltraPure Distilled Water (Invitrgen/GiBCO, Carlsbad, CA) and quantitated with the NanoDrop Spectrophotometer ND-1000 (Thermoscientific, Wilmington, DE), treated with DNase I (Invitrogen), then converted to cDNA using the Thermoscript RT PCR Reaction System (Invitrogen) according to the manufacture's protocol. The qRTPCR was conducted with human AR (forward: GACCAGATGGCTGTCATTCA and reverse: GGAGCCATCCAAACTCTTGA) and human GAPDH (forward: TGCACCACCAACTGCTTAGC and reverse: GGCATGGACTGTGGTCATGAG) primers in a Mastercycler ep realpex^2^ (eppendorf, Hamburg, Germany) using SYBR green PCR Master Mix (Applied Biosystems, Carlsbad, CA) to amplify the cDNA with the following PCR conditions; denatured at 95°C for 3 min and subjected to 40 cycles (95°C 30 sec, 60°C 30 sec, and 72°C 30 sec). The primers were used in a separate PCR and electrophoresed on a gel to verify the presence of a single amplicon from the cDNA. Each sample reaction in the qRTPCR was done in triplicate in a 96 well plate format. Cycle threshold units were obtained using Mastercycler ep realpex^2^ software. Data was analyzed using the 2^−ΔΔCT^ method [83] relative to GAPDH values. PCR was conducted on cDNA using the human AR and GAPDH primers referred to above in combination with platinum PCR super mix (Invitrogen). Reactions were run in an epindorf thermocycler denatured at 95°C for 3 min, subjected to 35 cycles (95°C 30 sec, 60°C 30 sec, and 72°C 30 sec) and processed on a 2% agarose gel.

### Chromatin Immunoprecipitation qPCR Analysis

For each ChIP 1×10^7^ BPH-1 cells were cross-linked with 1% formaldehyde for 10 min at room temperature on a rocking platform. The reaction was quenched with 0.125 M glycine. Cells were scraped and collected in cold PBS containing protease inhibitors (200 µg/ml PMSF, 50 µg/ml aprotinin, 5 µg/ml leupeptin, and 200 µM sodium orthovanidate), following 2 washes in cold PBS. The harvested cells were pelleted at 5,000 rpm for 6 min at 4°C and washed once with cold PBS containing protease inhibitors. Lysates were prepared using the reagents in the Magna ChIP A kit (Millipore, Temecula, CA) according to manufacture instructions, however, the lysis buffer available was substituted with 400 µl of SDS lysis buffer (Millipore) containing kit supplied protease inhibitors. The chromatin in the lysate was sheared to ≤600 bps in a 2 ml tube placed in a Covaris S2 (Covaris Inc., Woburn, MA) water bath set to the following cavitation parameters: duty cycle, 20%; intensity, 5; cycles per burst, 200; cycle time, 30 sec; and cycles, 30. The sheared chromatin was processed and immunoprecipitated with 5 µg of either DNMT1 (ab19505, abcam, Cambridge, MA) or rabbit IgG (sc-2027, Santa Cruz) using the reagents and instructions provided in the Magna ChIP A kit. The purified ChIP DNA was retrieved with 40 µl of elution buffer C. The DNA sample was amplified with a two step PCR program (Denaturation at 95°C for 10 min and 40 cycles of 95°C for 15 sec and 60°C for 1 min) using SYBR green PCR Master Mix in a StepOnePlus Real-Time PCR thermocycler (Applied Biosystems) employing the following primers; human E2F site A (forward: GACTCGCAAACTGTTGCATT and reverse: TACAGCACTGGAGCGGCTA), Site B (forward: CCTAGCAGGGCAGATCTTGT and reverse: TCCCCTTCTCTTGCTCAGAA), Site C (forward: GGTAGGAAGTGGCTGAATTCTGGATGA and reverse: CCCTGCCCATGCACCTGCTC), PS2 (forward: TTCCGGCCATCTCTCACTAT and reverse: CGGGGATCCTCTGAGACA), and ABCB1 (forward: TCTAGAGAGGTGCAACGGAAGCCA and reverse: CCTGCCCAGCCAATCAGCCT). An extended program (95°C for 15 min, 60°C for 1 min, and 95°C for 15 sec) was used to create a melting curve that was analyzed with the StepOne software v2.1 package to verify that the primers only amplify a single amplicon from genomic DNA. Each sample reaction in the qPCR was done in triplicate in a 96 well plate format. Cycle threshold units were obtained using StepOne software v2.1. Data is represented as a percent of input using a derivation of the 2^−ΔCT^ method [83].

### Bisulfite sequencing

Genomic DNA (gDNA) was extracted from cells using the Wizard Genomic DNA Purification kit (Promega) and quantified with the NanoDrop Spectrophotometer ND-1000 (Thermoscientific, Wilmington, DE). A 250 ng sample of DNA was bisulfite converted using the EZ DNA Methylation-Direct kit (Zymo Research, Orange, CA) according to the instructions provided by the manufacturer. The following bisulfite converted DNA specific primers, targeting a region in the AR (NM_000044) minimal promoter (forward: GGGAGTTAGTTTGTTGGGAG and reverse: TCCTACCAAACACTTTCCTTACT), were created with Methyl Primer Express v1.0. Amplification of the bisulfite converted gDNA was accomplished using special ZymoTaq PreMix (Zymo Research) polymerase to facilitate the production of amplicons with A overhangs using the following PCR program: denature at 95°C for 10 minutes, run 35 cycles (95°C 30 sec, 59°C 30 sec, and 72°C 60 sec), run a final extension at72°C for 7 min, and hold at 4°C. PCR product was combined with pCR8/GW/TOPO TA cloning vector (Invitrogen) in a mixture prescribed by the manufacture to facilitate the insertion of the amplified products into the plasmids which contain sequencing primer sites that flank the insert. Plasmids were transformed and plated in One Shot Top 10 chemically competent cells (Invitrogen) per manufacturer's instructions and at least 12 bacterial colonies were individually grown in 5 ml of LB containing spectinomycin (100 µg/ml). Plasmids were harvested from the bacteria using the Wizard Plus SV Miniprep kit (Promega) and sequenced with the M13 forward and reverse primers at the University of Michigan DNA sequencing core.

### Statistics

Data showing significance was analyzed using 2-tailed Student's t test. P<0.05 was accepted as the level of significance.
